# Validation of the preconfigured Varian Ethos Acuros XB Beam Model for treatment planning dose calculations: A dosimetric study

**DOI:** 10.1002/acm2.13056

**Published:** 2020-10-17

**Authors:** Yunfei Hu, Mikel Byrne, Ben Archibald‐Heeren, Nick Collett, Guilin Liu, Trent Aland

**Affiliations:** ^1^ Icon Cancer Center Gosford Gosford NSW Australia; ^2^ Icon Cancer Centre Wahroonga Wahroonga NSW Australia; ^3^ Icon Core Office South Brisbane QLD Australia

**Keywords:** Acuros XB, adaptive, beam model, Ethos, validation

## Abstract

Varian (Palo Alto, California, United States) recently released an online adaptation treatment platform, Ethos, which has introduced a new Dose Preview and Automated Plan Generation module despite sharing identical beam data with the existing Halcyon linac. The module incorporates a preconfigured beam model and the Acuros XB algorithm (Ethos AXB model) to generate final dose calculations from an initial fluence optimization. In this study, we comprehensively validated the accuracy of the Ethos AXB model by comparing it against the Halcyon AXB model, the Halcyon Anisotropic Analytical Algorithm (AAA) model, and measurements acquired on an Ethos linac. Results indicated that the Ethos AXB model demonstrated a comparable if not superior dosimetric accuracy to the Halcyon AXB model in basic and complex calculations, and at the same time its dosimetric accuracy in modulated and heterogeneous plans was better than that of the Halcyon AAA model. Despite the fact that the same algorithm was utilized, the Ethos AXB model and the Halcyon AXB model still exhibited variations across a range of tests, although these variations were predominantly insignificant in the clinical environment. The accuracy of the Ethos AXB model has been successfully verified in this study and is considered appropriate for the current clinical scope. On the basis of this study, clinical physicists can perform a data validation instead of a full data commissioning when implementing the Ethos system, thereby adopting a more efficient approach for Ethos installation.

## 1 INTRODUCTION

Varian (Palo Alto, California, United States) recently released an on‐line adaptive treatment platform, Ethos, which allows on‐couch plan adaptation and treatment monitoring.^1^ Although the Ethos system is a conversion of the existing Halcyon linac, it has introduced novel computer hardware and software to enable adaptive radiotherapy, including a Dose Preview and Automated Plan Generation module that generates plans and computes doses automatically. This module utilizes the following algorithms^2^:

‐ Intelligent Optimization Engine
‐ Photon Optimization algorithm for volumetric modulated arc therapy (VMAT) and intensity modulated radiotherapy (IMRT)
‐ Smart LMC algorithm for leaf sequencing in IMRT
‐ DVH Estimation algorithm for DVH estimation
‐ Fourier Transform Dose Calculation (FTDC) for dose calculation during optimization
‐ Acuros XB (AXB) for final dose calculation, and for calculation of intermediate dose during optimization.

While different algorithms are used at different stages of plan generation, the final dose calculation in Ethos is performed by an implementation of the AXB algorithm previously introduced in another treatment planning system (TPS), Varian Eclipse. Although the accuracy of the AXB algorithm in the Eclipse TPS has been verified by multiple studies,^3^, ^4^, ^5^, ^6^, ^7^, ^8^ it has been implemented into a dedicated environment (the Ethos TPS) using dedicated computing hardware. Previous investigations between AXB calculations with central processing unit (CPU) and graphical processing units (GPU) demonstrate the accuracy of AXB with GPU calculations,^9^ however, no such testing exists in the literature for the Ethos platform.

Varian has introduced a preconfigured beam model for Ethos and Halcyon, which is created based on the 6 MV FFF golden beam data (GBD) shared by both linacs. When a new algorithm or a new beam model is introduced in a clinical practice, tests should be performed to appraise and analyze the inherent physical approximations of the model and their adequacy to reproduce dosimetric data relevant for clinical usage.^10^, ^11^ The use of different software and hardware between Ethos and Halcyon can potentially introduce variations in the derived beam models and their performance. Therefore, it is users’ responsibility to verify the accuracy of the preconfigured Ethos AXB beam model in the dedicated Ethos TPS.

The purpose of this study was to validate the preconfigured Varian Ethos AXB model for treatment planning dose calculations. For this purpose, comparisons were conducted between measurements and dose calculations performed with each of the following beam models:

‐ The Ethos model and the AXB algorithm (Ethos AXB model),
‐ The Eclipse Halcyon 2.0 model and the AXB algorithm (Halcyon AXB model),
‐ The Eclipse Halcyon 2.0 model and the Anisotropic Analytical Algorithm (AAA) algorithm (Halcyon AAA model).

In this study, both the Halcyon AXB model and the Halcyon AAA model were compared to the Ethos AXB model. The Halcyon AXB Model was chosen because it uses the same algorithm (AXB) with the Ethos TPS, and its implementation in the Eclipse environment has been extensively verified.^3^, ^4^, ^5^, ^6^, ^7^, ^8^ Calculations were also compared to the Halcyon AAA model, because its accuracy was not only verified by authors during the initial commissioning but has also been validated in several other studies.^12^, ^13^, ^14^ A similar approach was adopted by a previous study during the validation of the Eclipse AXB algorithm.^15^ All calculation results were also compared to measurements acquired on an Ethos linac.

## 2 MATERIALS AND METHODS

The AXB algorithm requires the macroscopic atomic cross sections of the components of the dose calculation material.^16^, ^17^ Therefore, the authors established a calibration curve that converted Hounsfield units to mass densities in the Ethos TPS by scanning a CIRS (Norfolk, Virginia, United States) CT‐ED phantom. Subsequently, the algorithm determines the material composition from the voxels in the image by a predefined material library.^17^

To verify the accuracy of the Ethos AXB model under different clinical scenarios, three levels of tests were created, including:

- basic tests for a fundamental dosimetric characterization of the algorithm;
- advanced tests for specific planning or treatment conditions; and
- advanced tests to investigate management of tissue heterogeneity in the new algorithm.^11^, ^18^

Each test was respectively calculated using the Ethos AXB model in Ethos TPS v1.0, the Halcyon AXB model in Eclipse v15.6, and the Halcyon AAA model in Eclipse v15.6. Calculations in the Ethos TPS were performed using the smallest resolution available, which was 2.5 mm. Alternatively, for the Eclipse TPS, PDD and profile calculations were performed with the smallest resolution, which was 1.0 mm, while all other calculations (output factors and advanced tests) were performed with the clinically used resolution, which was 2.5 mm.

These calculations were subsequently compared to measurements using appropriate detectors (various ionization chambers for point dose measurements and SunNuclear (Melbourne, Florida, United States) ArcCheck for 3D measurements). Measurements were acquired on an Ethos linac converted from a Halcyon linac which the Halcyon AXB and AAA models were created for. The beam data of the Ethos linac were tuned to match the GBD supplied by Varian, thereby making it possible to compare measurements to both the Ethos AXB model and the Halcyon models.

Specific plans included in each level of test consisted of:

‐ Basic tests: PDDs and profiles of standard square fields; output factors of standard square fields; and output factors of elongated fields.
‐ Advanced tests for specific planning or treatment conditions: chair test with varying modulation; and clinical plans of anatomical sites in solid water slabs (SunNuclear, Florida) and the cylindrical ArcCheck array detector (SunNuclear, Florida)
‐ Advanced tests to investigate the management of tissue heterogeneity: clinical plans of various anatomical sites in an inhomogeneous CIRS thorax phantom (Model No. 002LFC).

Ethos utilizes a dual‐multi‐leaf‐collimator (MLC) design that has a maximum square field size of 28 × 28 cm^2^. Therefore, in this study, all verification tests were limited to field sizes smaller than or equal to 28 × 28 cm^2^. All fields measured in this study were defined by MLCs as the machine does not have jaws. All comparisons were conducted using the 6 MV flattening filter‐free (FFF) energy, the only available energy on both Ethos and Halcyon linacs.

### 2.A Basic tests

Treatment fields for basic tests were created in Eclipse in a 40 × 40 × 40 cm^3^ virtual water tank. Each field was computed using the Halcyon AXB and AAA models, respectively. Subsequently, the plan and its associated structures and datasets were exported to the Ethos TPS to recalculate using the Ethos AXB model. The calculation results were then compared to measurements.

#### 2.A.1 PDDs and profiles of standard square fields

PDDs were acquired using a PTW (Freiburg, Germany) Semiflex 3D ionization chamber for fields ≥ 4 cm^2^ and a PTW microdiamond^19^ in the edge‐on orientation^20^ for fields <4 cm^2^ in a PTW Beamscan water tank under the following field sizes: 1 × 1, 2 × 2, 4 × 4, 10 × 10, and 28 × 28 cm^2^. Profiles were acquired using the same detector and water tank, but only under field sizes of 4 × 4, 10 × 10 and 28 × 28 cm^2^ and depths of 1.3 cm (D_max_), 10.0 and 20.0 cm. The source‐to‐surface distances (SSDs) of all measurements were 90 cm.

#### 2.A.2 Output factors of standard square fields

For standard square fields, output factors were collected under the following field sizes: 1 × 1, 2 × 2, 3 × 3, 4 × 4, 6 × 6, 8 × 8, 10 × 10, 14 × 14, 20 × 20, and 28 × 28 cm^2^. Measurements were taken with a PTW Semiflex 3D ionization chamber for fields ≥ 4 cm^2^ and a microdiamond in the edge‐on orientation for fields < 4 cm^2^, with the detector sitting at 10 cm depth and 90 cm SSD in a water tank. Readings were referenced back to that of 10 × 10 cm^2^ through daisy‐chaining^21^ to calculate output factors.

#### 2.A.3 Output factors of elongated fields

Output factors of elongated fields were acquired under the following field sizes: 3 × 28, 4 × 16, 5 × 20, 10 × 15, 15 × 10, 20 × 5, 16 × 4, and 28 × 3 cm^2^. Measurements were taken with a PTW Semiflex 3D ionization chamber, with the chamber sitting at 5 cm depth and 95 cm SSD in a water tank. Readings were referenced back to that of 10 × 10 cm^2^ to calculate output factors.

### 2.B Advanced tests for specific planning or treatment conditions

There are two tests in this section: a set of chair tests with increasing modulation, and a set of clinical plans. Both sets of plans were measured in homogeneous phantoms.

#### 2.B.1 Chair tests

A set of five chair test plans were optimized in a homogenous water volume. Modulation characteristics of each plan were controlled by implementing incremental X and Y smoothing parameters for IMRT optimization. Smoothing parameters ranged from X = 10, Y = 20 to X = 60, Y = 70, respectively. Each plan was optimized with the same dose objectives for the chair‐shaped target and surrounding avoid structures. The increase of modulation in each of the respective plans was evaluated by total plan MU per target dose (MU/Gy).

The plans were subsequently delivered to homogeneous solid water slabs, in which a PTW Semiflex 3D chamber was inserted. The measured chamber reading was then compared to the calculated point dose at the corresponding position.

#### 2.B.2 Clinical plans in a homogeneous phantom

A set of clinical plans of different anatomical sites, including abdomen, brain, bilateral head and neck, unilateral head and neck, and prostate, were retrospectively selected and recomputed on the ArcCheck phantom in both TPSs. In both systems, the density of the ArcCheck was overridden to “Poly(methyl methacrylate) (PMMA)” (1.19 g/cm^3^). Once computed, plans were delivered to ArcCheck, followed by comparing the measured and the calculated dose distributions in the SunNuclear SNC Patient software. In addition, a comparison was also performed directly between the three models. The gamma criteria used for analysis were 3% and 2 mm.^22^ During measurements, a PTW Semiflex 3D chamber was inserted at the isocenter of the ArcCheck to measure point doses.

### 2.C Advanced tests to investigate the management of tissue heterogeneity

**Fig. 1:**
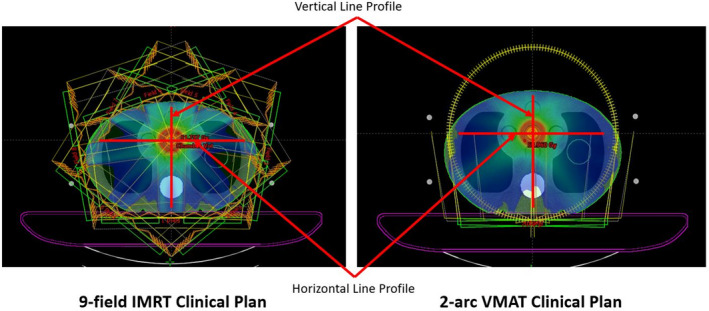
An illustration of the IMRT and VMAT clinical plan dose distributions. A vertical line dose profile (going through the target and the bone) and a horizontal line dose profile (going through the target and both lungs) were plotted and compared between the two models.

In this section, two clinical plans, a VMAT and an IMRT plan, were computed in both TPSs on a CIRS thorax phantom, which consisted of tissue, lung, and bone materials. A Standard Imaging (Middleton, Wisconsin, United States) Exradin A1SL chamber was used to take measurements at the center of the phantom and compare to the calculation results. In both plans, the beam arrangement meant that most of the dose was delivered through significant heterogeneity, as shown in Fig. 1.

Phantom images used in planning were scanned with the chamber insert, and therefore the position of the chamber was air in these images. AXB explicitly models the physical interactions of radiation and matter, whereas AAA use pre‐calculated MC kernels scaled according to local density variations.^16^ In addition, the Ethos TPS can only report dose to medium (D_m_), which in this case would report dose in air. However, when appropriate correction factors are applied, chambers measure dose to water (D_w_). In order to compare calculated and measured doses, in both the Ethos TPS and the Eclipse TPS, the chamber hole was overridden to the material of water (1.0 g/cm^3^). In addition, as Ethos does not allow the report of per beam dose, for both the IMRT and the VMAT plans, integral plan doses were instead reported and compared.

In addition to point doses, a vertical line dose profile (going through the target and the bone) and a horizontal line dose profile (going through the target and both lungs) were plotted and compared between the Ethos AXB model and the Halcyon models.

### 2.D Statistical analysis and uncertainty

Where statistical analysis was required, a one‐tailed t‐test was performed provided the number of samples was large enough. *P* < 0.05 was considered statistically significant.

For chamber measurements that were acquired together with an electrometer, the uncertainty was calculated using the formula below:

$$\varepsilon_{\mathrm{total}}=\sqrt{\varepsilon_{a}^{2}+\varepsilon_{b}^{2}},$$

where ɛ_a_ and ɛ_b_ are the estimated measurement uncertainty of each component (chamber and electrometer) in the measurement system. In this paper, three chambers were used during measurements, which were: PTW microdiamond, Semiflex 3D, and Exradin A1SL. The individual long‐term stability of the PTW microdiamond and Semiflex 3D detector was 0.25%^23^ and 0.30%^24^ according to their technical specifications. The long‐term stability of the Exradin A1SL chamber was not specified by the vendor but was estimated to be 0.5% based on authors' experience with the chamber. For all measurements, a PTW Unidos Webline electrometer was used, the uncertainty of which was 0.5%^.^ ^25^ Therefore, using the above formula, the combined uncertainty of the PTW microdiamond, the Semiflex 3D, and the Exradin A1SL measurement system was calculated to be 0.6%, 0.6%, and 0.7%, respectively. As imaging was used in all measurement setups, setup uncertainty was considered negligible and therefore excluded in the uncertainty analysis.

## 3 RESULTS

### 3.A Basic tests

#### 3.A.1 PDDs and profiles of standard square fields

**Fig. 2:**
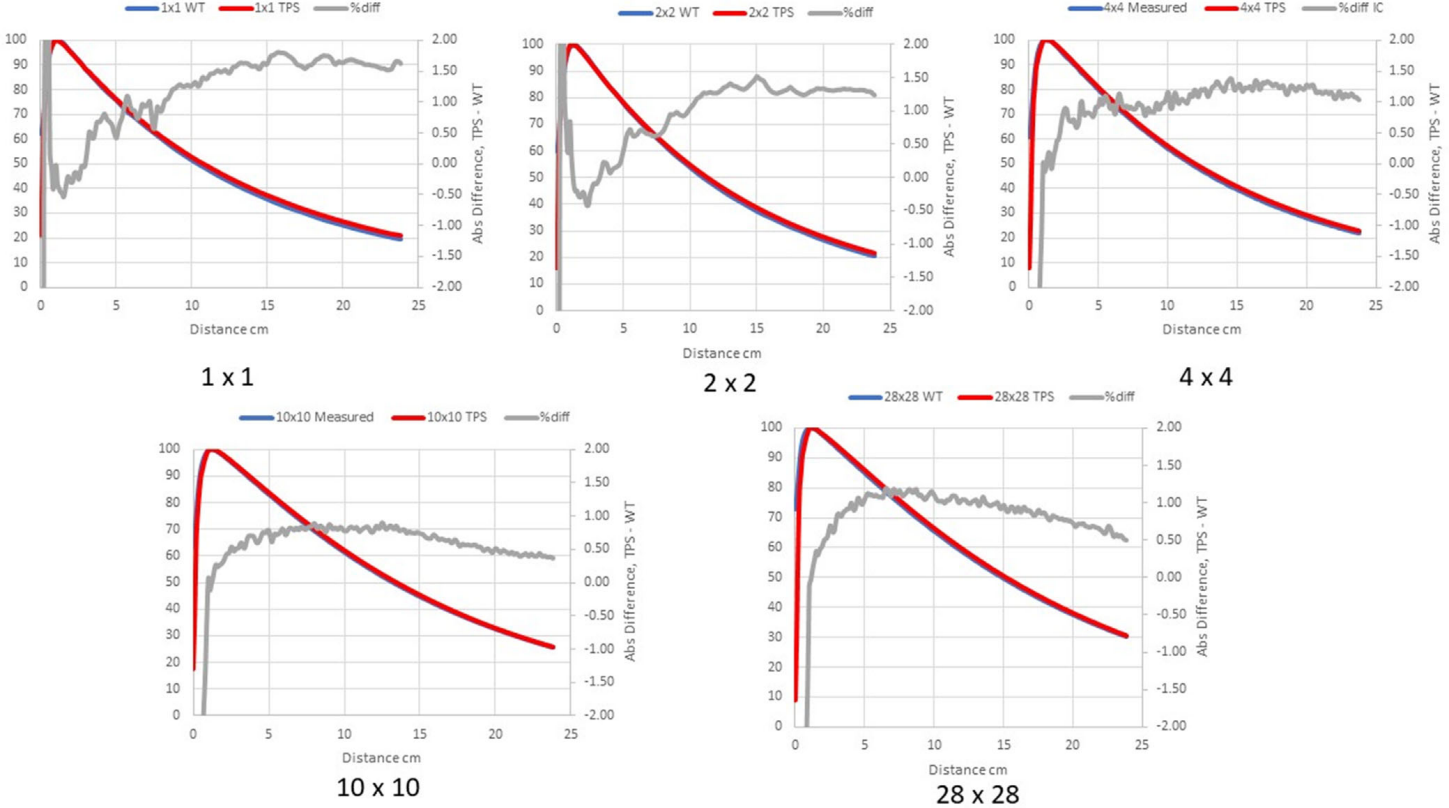
Comparison of measured PDDs and PDDs calculated with the Ethos AXB model. Red: Calculated PDD; Blue: Measured PDD; Grey: % difference.

**Fig. 3:**
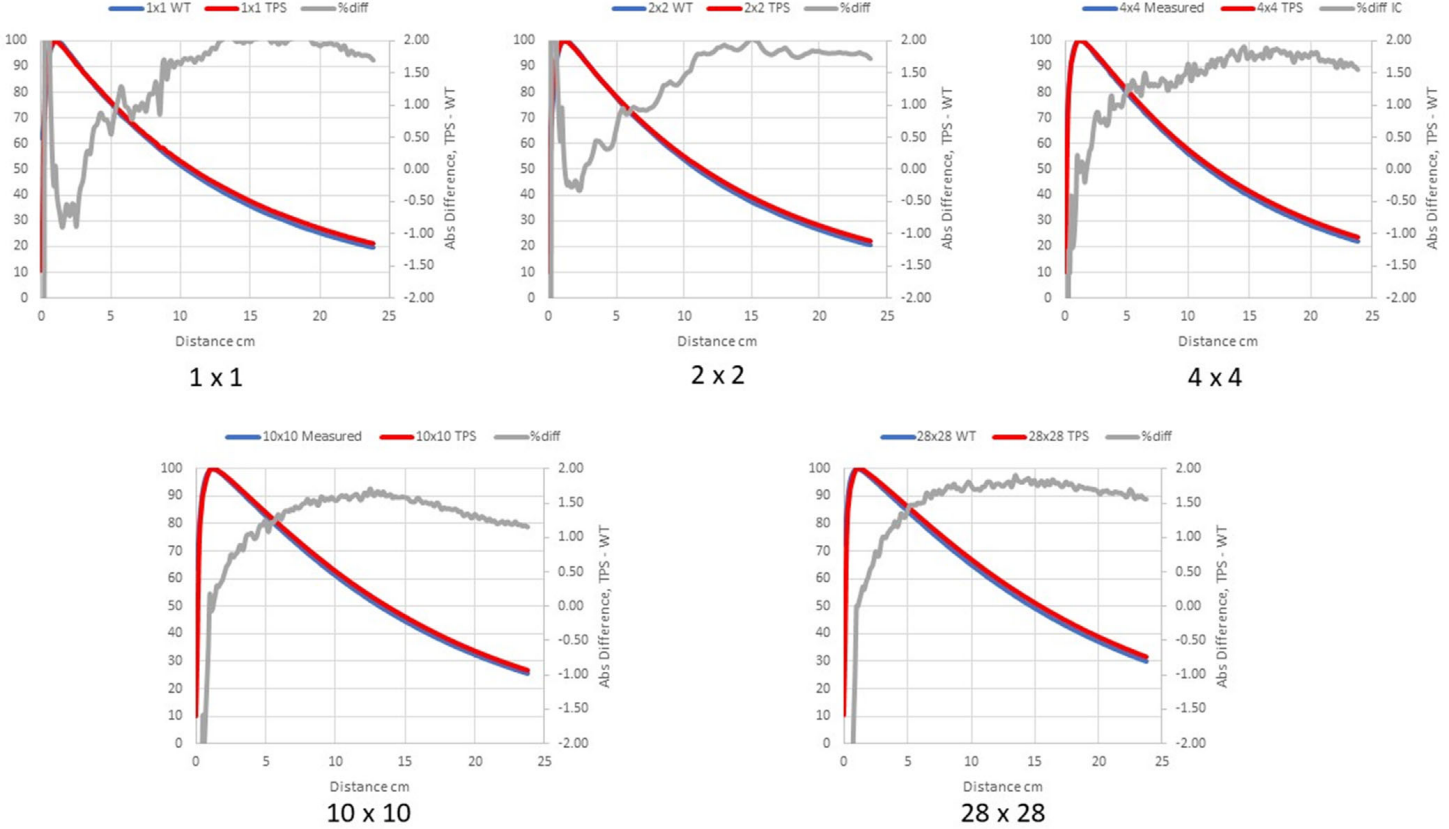
Comparison of measured PDDs and PDDs calculated with the Halcyon AXB model. Red: Calculated PDD; Blue: Measured PDD; Grey: % difference.

**Fig. 4:**
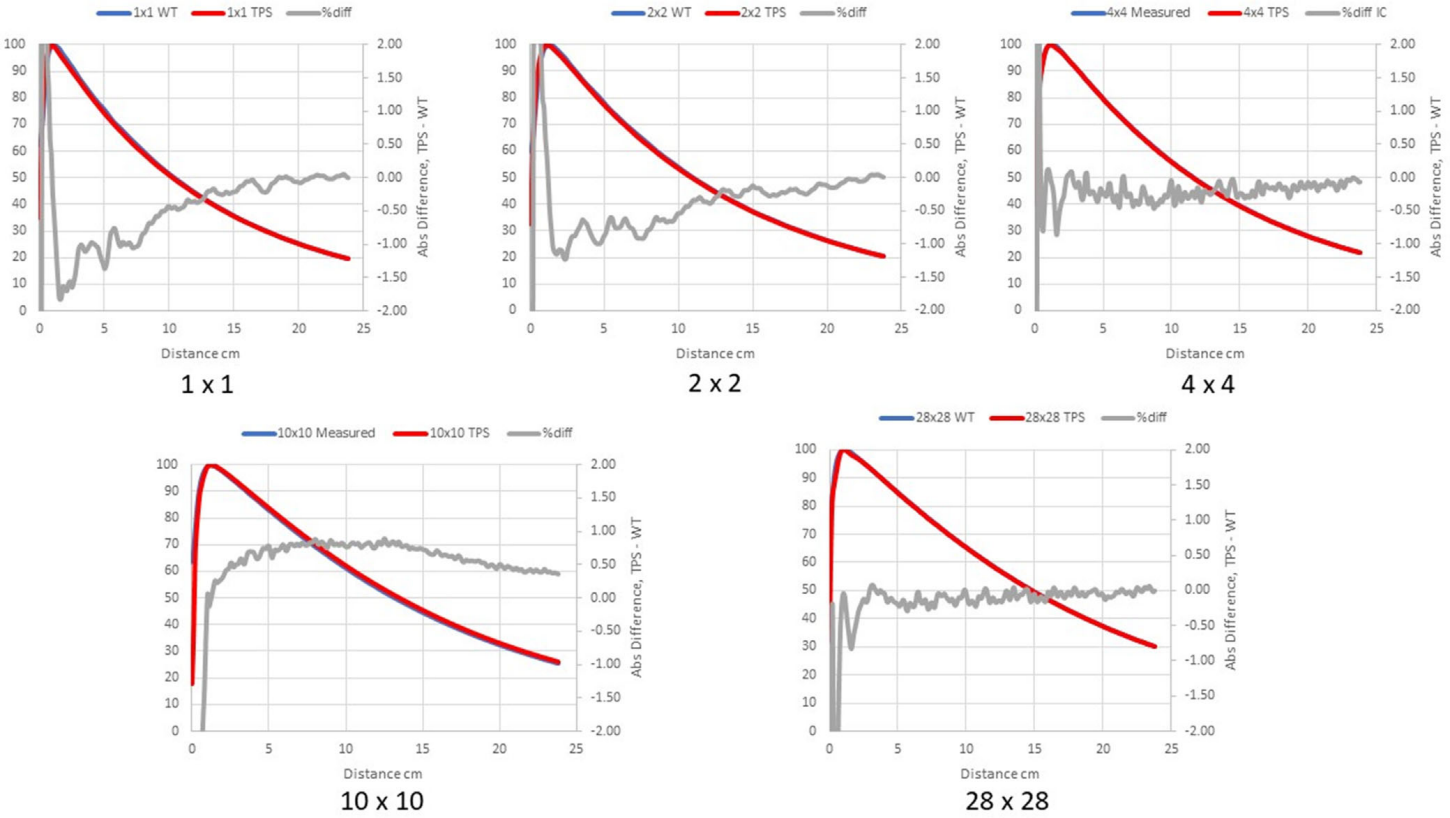
Comparison of measured PDDs and PDDs calculated with the Halcyon AAA model. Red: Calculated PDD; Blue: Measured PDD; Grey: % difference.

**Fig. 5:**
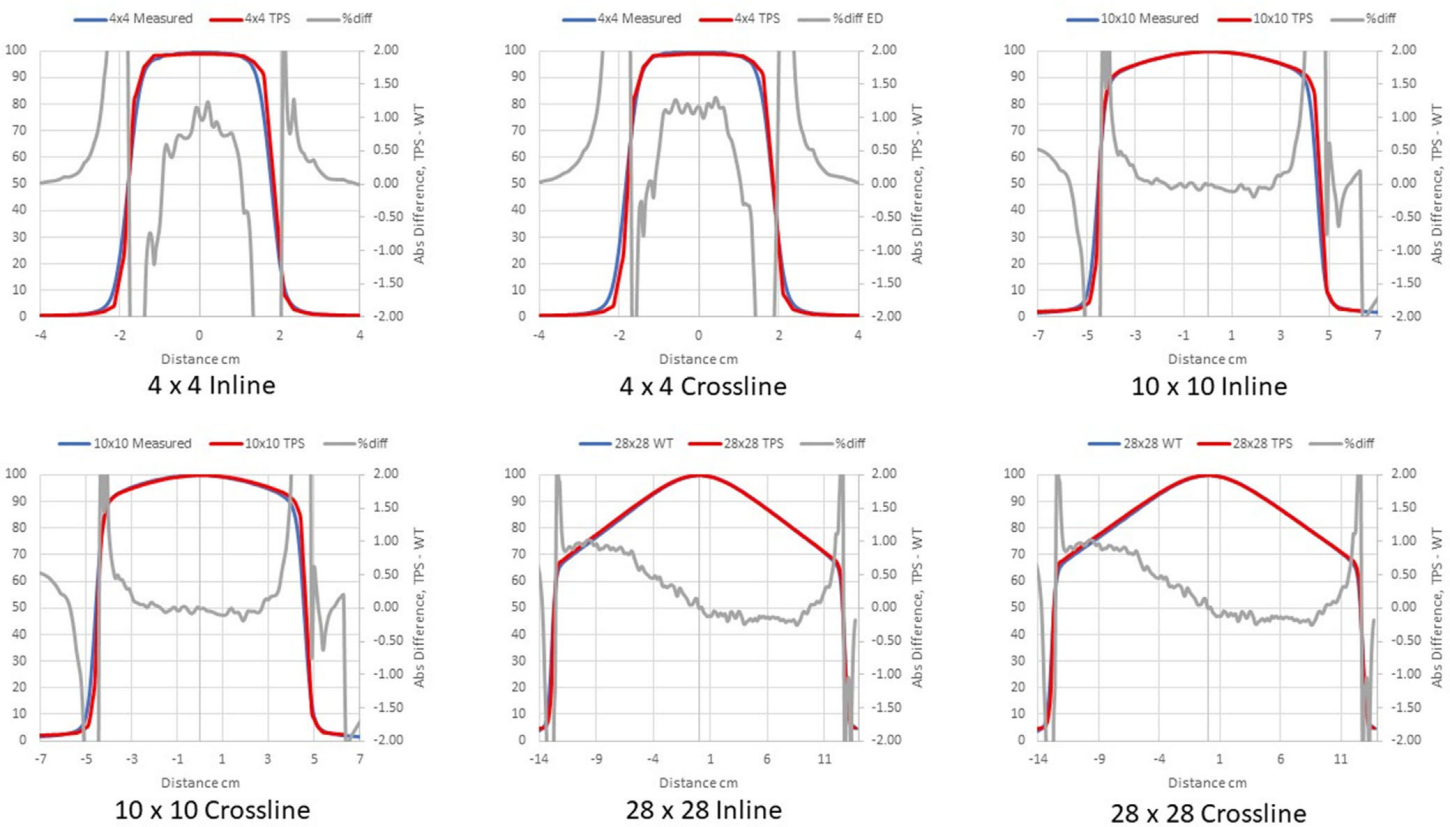
Comparison of measured profiles and profiles calculated with the Ethos AXB model. Only profiles measured at a depth of 1.3 cm were displayed in the figure. Red: Calculated profile; Blue: Measured profile; Grey: % difference.

**Fig. 6:**
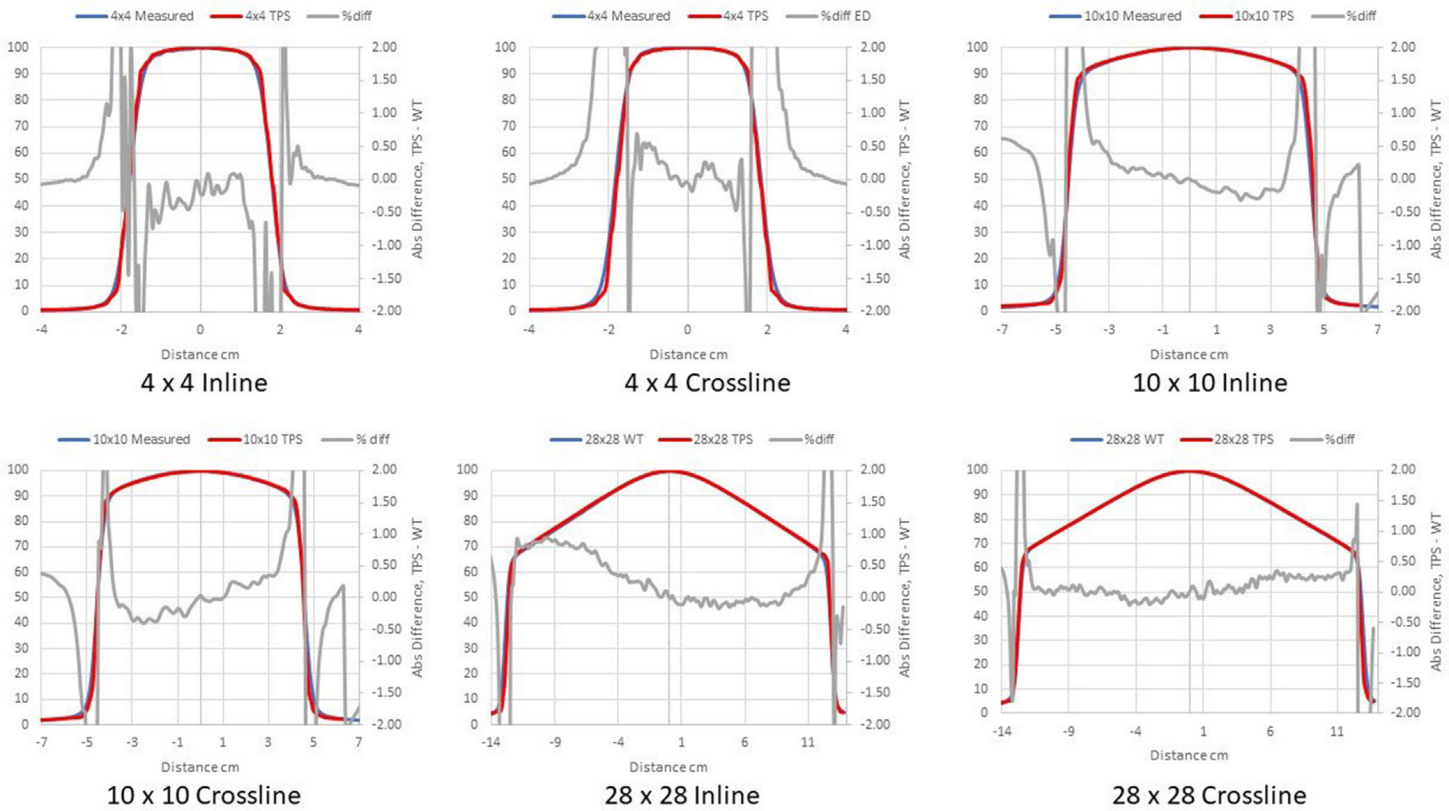
Comparison of measured profiles and profiles calculated with the Halcyon AXB model. Only profiles measured at a depth of 1.3 cm were displayed in the figure. Red: Calculated profile; Blue: Measured profile; Grey: % difference.

**Fig. 7:**
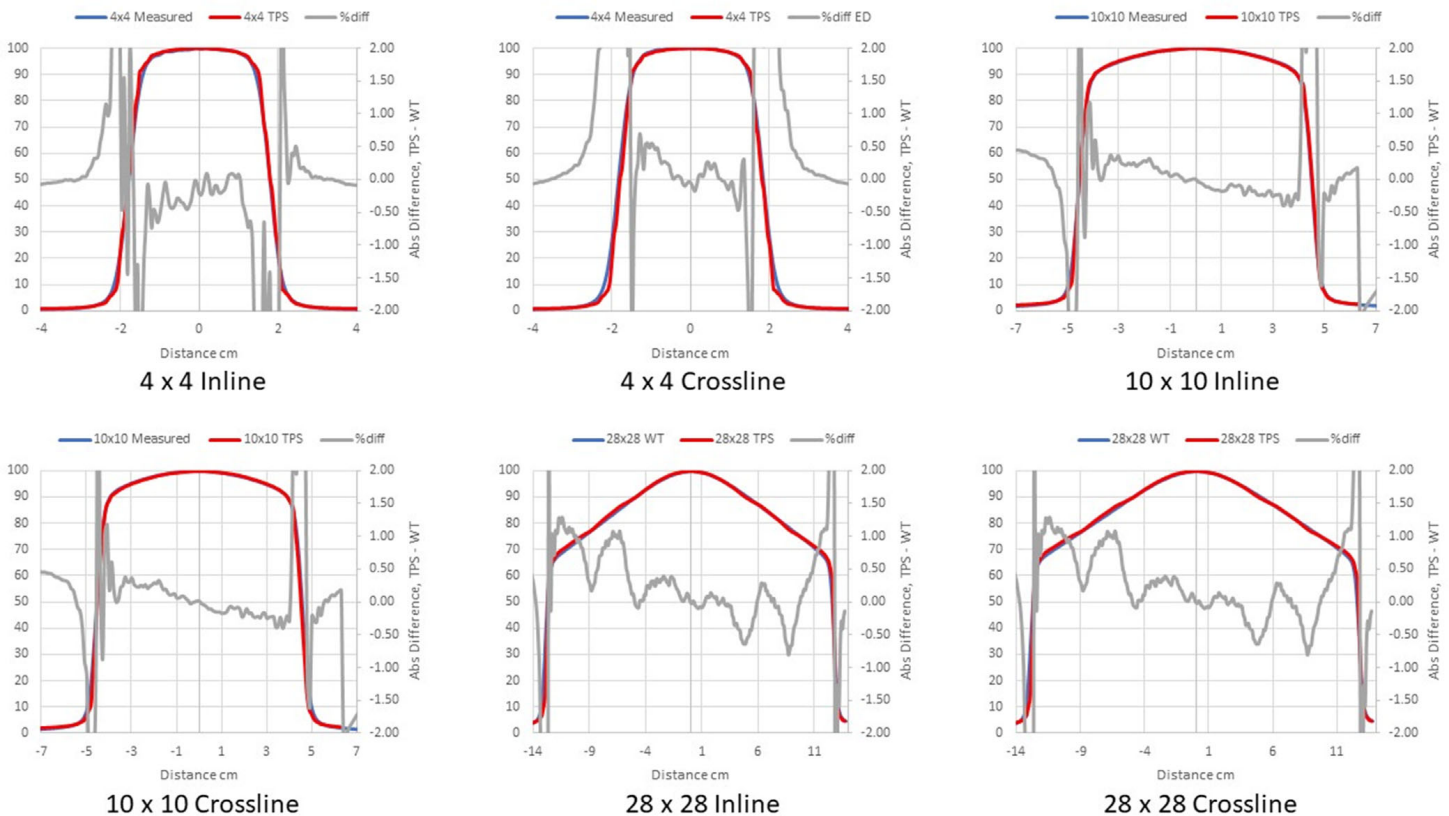
Comparison of measured profiles and profiles calculated with the Halcyon AAA model. Only profiles measured at a depth of 1.3 cm were displayed in the figure. Red: Calculated profile; Blue: Measured profile; Grey: % difference.

Figures 2, 3, 4 compare measured PDDs with PDDs calculated by the Ethos AXB model, the Halcyon AXB model, and the Halcyon AAA model, respectively. Figures 5, 6, 7 compare measured profiles with profiles calculated by the Ethos AXB model, the Halcyon AXB model, and the Halcyon AAA model, respectively.

#### 3.A.2 Output factors of standard square fields

Table 1 lists measured output factors of standard square fields as well as output factors calculated by the Ethos AXB model, the Halcyon AXB model, and the Halcyon AAA model, respectively.

**Table 1:** Output factors of standard square fields.

Field size (cm^2^)	Measured output factor	Ethos AXB		Halcyon AXB		Halcyon AAA	
		Output factor	Difference (%)	Output factor	Difference (%)	Output factor	Difference (%)
1 × 1	0.702^a^	0.701	−0.1%	0.701	−0.2%	0.695	−1.0%
2 × 2	0.805^a^	0.798	−0.9%	0.798	−0.8%	0.791	−1.7%
3 × 3	0.847^a^	0.846	−0.1%	0.846	−0.2%	0.841	−0.7%
4 × 4	0.880^b^	0.879	−0.1%	0.879	−0.1%	0.878	−0.2%
6 × 6	0.929^b^	0.929	0.0%	0.929	0.0%	0.941	1.3%
8 × 8	0.969^b^	0.969	0.0%	0.969	0.0%	0.969	0.0%
10 × 10	1.000^b^	1.000	0.0%	1.000	0.0%	1.000	0.0%
14 × 14	1.044^b^	1.045	0.1%	1.045	0.1%	1.047	0.3%
20 × 20	1.087^b^	1.087	0.0%	1.087	0.0%	1.088	0.1%
28 × 28	1.115^b^	1.116	0.1%	1.116	0.1%	1.116	0.1%

^a^ Measurement uncertainty = 0.6%.

^b^ Measurement uncertainty = 0.6%.

#### 3.A.3 Output factors of elongated fields

Table 2 lists measured output factors of elongated fields as well as output factors calculated by the Ethos AXB model, the Halcyon AXB model, and the Halcyon AAA model, respectively.

**Table 2:** Output factors of elongated fields.

Field size (cm^2^)	Measured output factor	Ethos AXB		Halcyon AXB		Halcyon AAA	
		Output factor	Difference (%)	Output factor	Difference (%)	Output factor	Difference (%)
3 × 28	0.946^a^	0.938	−0.9%	1.000	−1.2%	0.936	−1.0%
4 × 16	0.958^a^	0.958	0.0%	0.935	−0.2%	0.956	−0.2%
5 × 20	0.978^a^	0.976	−0.2%	0.957	−0.3%	0.974	−0.4%
10 × 15	1.013^a^	1.014	0.1%	0.975	0.0%	1.013	0.0%
15 × 10	1.014^a^	1.015	0.1%	1.013	−0.1%	1.014	0.0%
20 × 5	0.975^a^	0.979	0.4%	1.013	0.1%	0.974	−0.1%
16 × 4	0.960^a^	0.959	−0.1%	0.976	−0.4%	0.956	−0.4%
28 × 3	0.936^a^	0.938	0.2%	0.957	−0.5%	0.933	−0.3%

^a^ Measurement uncertainty = 0.6%.

### 3.B Advanced tests for specific planning or treatment conditions

#### 3.B.1 Chair tests

Table 3 lists measured point doses of chair tests with different modulations as well as those calculated by the Ethos AXB model, the Halcyon AXB model, and the Halcyon AAA model, respectively.

**Table 3:** Central point doses of chair tests with different modulations.

Plan No.	MU/Gy	Measured output factor	Ethos AXB		Halcyon AXB		Halcyon AAA	
			Output factor	Difference (%)	Output factor	Difference (%)	Output factor	Difference (%)
1	527.2	2.133^a^	2.153	1.0%	2.143	0.5%	2.183	2.3%
2	429.4	2.133^a^	2.154	1.0%	2.125	−0.4%	2.115	−0.8%
3	340.9	2.136^a^	2.156	0.9%	2.146	0.4%	2.177	1.9%
4	309.0	2.173^a^	2.159	0.8%	2.132	−0.4%	2.201	1.3%
5	279.8	2.142^a^	2.185	0.6%	2.163	−0.5%	2.179	1.8%

^a^ Measurement uncertainty = 0.6%.

#### 3.B.2 Clinical plans in a homogeneous phantom

Table 4 lists measured point doses of various clinical plans as well as those calculated by the Ethos AXB model, the Halcyon AXB model, and the Halcyon AAA model, respectively.

**Table 4:** Clinical plan chamber measurement results.

Plan type	Measured dose (Gy)	Ethos AXB		Halcyon AXB		Halcyon AAA	
		Calculated dose (Gy)	Difference (%)	Calculated dose (Gy)	Difference (%)	Calculated dose (Gy)	Difference (%)
Abdomen	2.08^a^	2.08	0.0%	2.08	0.0%	2.09	0.6%
Brain	1.77^a^	1.76	−0.8%	1.78	0.6%	1.78	0.4%
Bilateral Head and Neck	1.87^a^	1.89	0.9%	1.89	0.9%	1.90	1.4%
Unilateral Head and Neck	1.54^a^	1.54	−0.4%	1.53	−0.6%	1.53	−0.7%
Prostate	2.43^a^	2.42	−0.6%	2.42	−0.5%	2.43	0.0%
Breast IMRT	1.66^a^	1.70	2.5%	1.70	2.6%	1.71	3.1%

^a^ Measurement uncertainty = 0.6%.

Table 5 lists the ArcCheck 3 mm/2% gamma pass rates of the Ethos AXB model, the Halcyon AXB model, and the Halcyon AAA model when compared to measurements. In addition, the calculated ArcCheck plans of the Ethos AXB model, the Halcyon AXB model, and the Halcyon AAA model were directly compared, the results of which are shown in Table 6.

**Table 5:** Clinical plan ArcCheck gamma pass rates (3 mm/2%)

Plan type	Ethos AXB vs measurement	Halcyon AXB vs measurement	Halcyon AAA vs measurement
Abdomen	100.0%	100.0%	100.0%
Brain	99.9%	99.9%	99.9%
Bilateral Head and Neck	100.0%	100.0%	100.0%
Unilateral Head and Neck	99.7%	99.5%	99.2%
Prostate	100.0%	100.0%	100.0%
Breast IMRT	100.0%	100.0%	99.8%

**Table 6:** Gamma pass rates (3 mm/2%) of direct comparisons between ArcCheck plans calculated by different models.

Plan type	Ethos AXB vs Halcyon AXB	Ethos AXB vs Halcyon AAA	Halcyon AXB vs Halcyon AAA
Abdomen	100.0%	100.0%	100.0%
Brain	100.0%	100.0%	100.0%
Bilateral Head and Neck	100.0%	100.0%	100.0%
Unilateral Head and Neck	100.0%	100.0%	100.0%
Prostate	100.0%	100.0%	100.0%
Breast IMRT	100.0%	100.0%	100.0%

### 3.C Advanced tests to investigate the management of tissue heterogeneity

Table 7 lists measured point doses of two clinical plans (IMRT and VMAT) with heterogeneity as well as those calculated by the Ethos AXB model, the Halcyon AXB model, and the Halcyon AAA model, respectively.

**Table 7:** Clinical plan heterogeneity chamber measurement results.

Plan type	Measured dose (Gy)	Ethos AXB		Halcyon AXB		Halcyon AAA	
		Calculated dose (Gy)	Difference (%)	Calculated dose (Gy)	Difference (%)	Calculated dose (Gy)	Difference (%)
IMRT	2.08^a^	2.06	−0.8%	2.07	−0.5%	2.05	−1.2%
VMAT	2.07^a^	2.04	−1.6%	2.04	−1.6%	2.03	−2.0%

^a^ Measurement uncertainty = 0.7%.

**Fig. 8:**
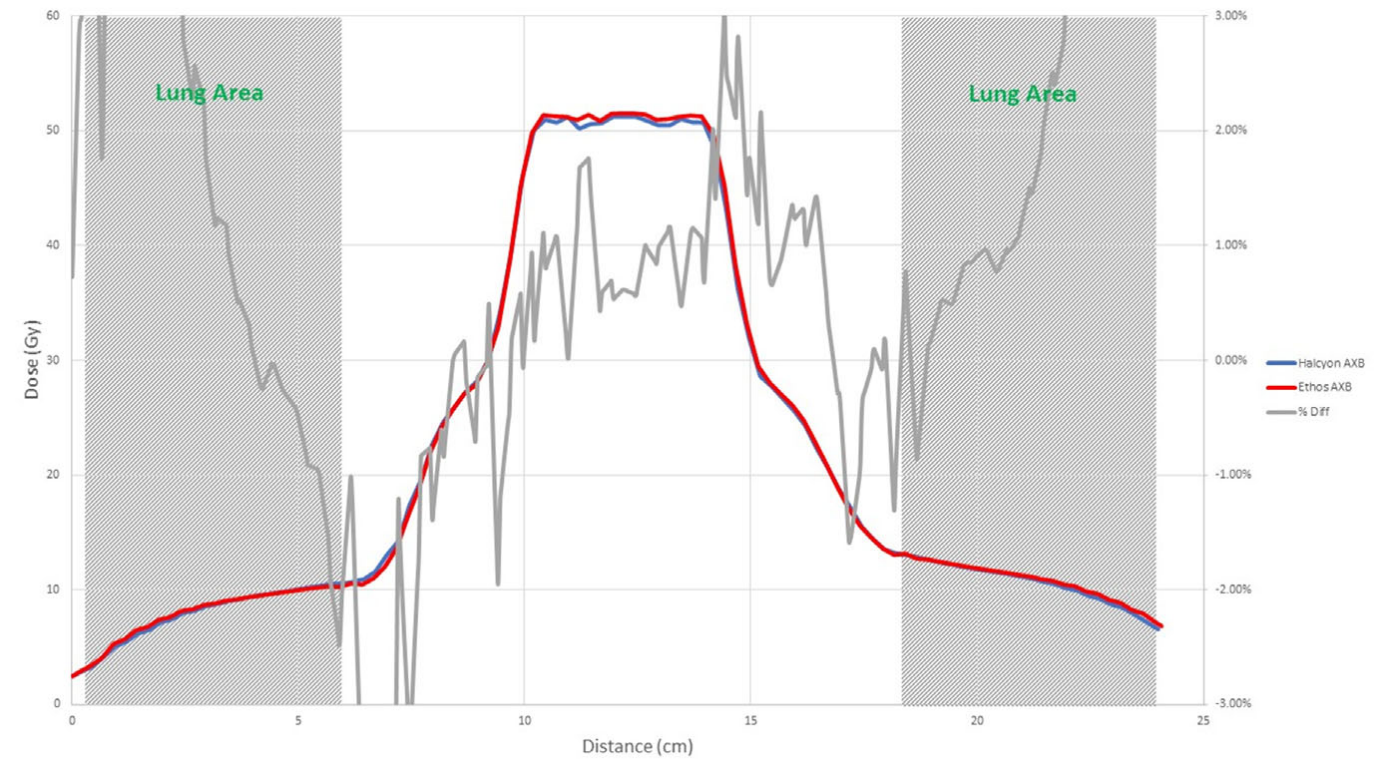
Comparison of the horizontal line dose profile of the IMRT plan calculated by the Ethos AXB model and the Halcyon AXB model.

**Fig. 9:**
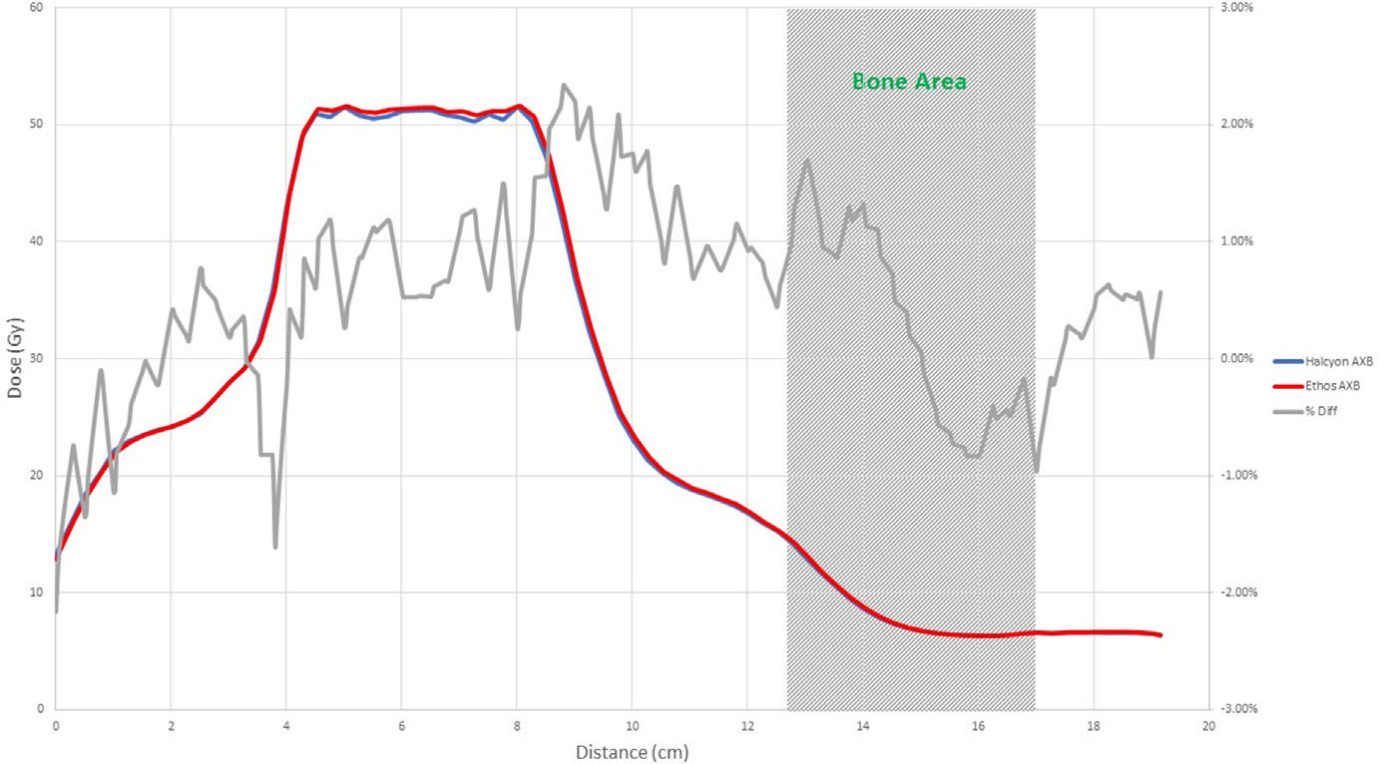
Comparison of the vertical line dose profile of the IMRT plan calculated by the Ethos AXB model and the Halcyon AXB model.

**Fig. 10:**
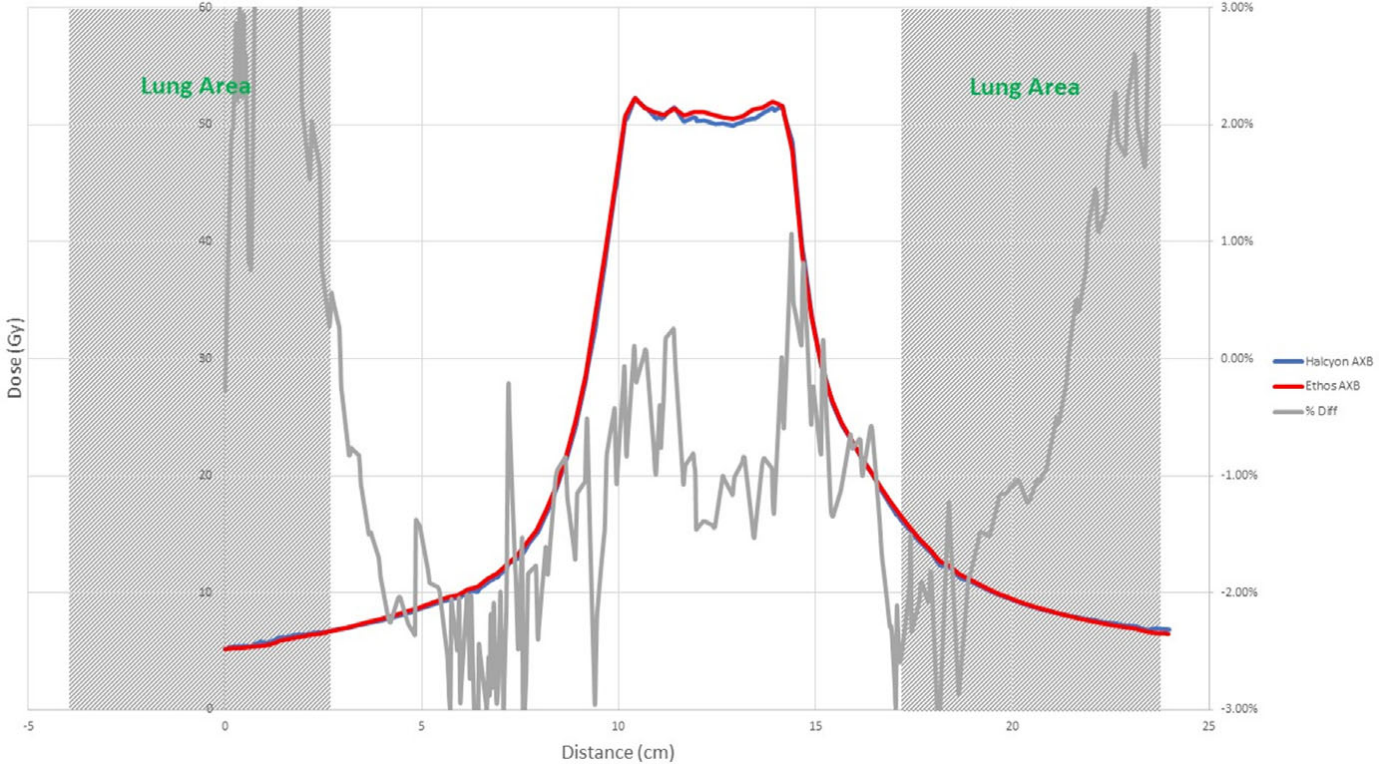
Comparison of the horizontal line dose profile of the VMAT plan calculated by the Ethos AXB model and the Halcyon AXB model.

**Fig. 11:**
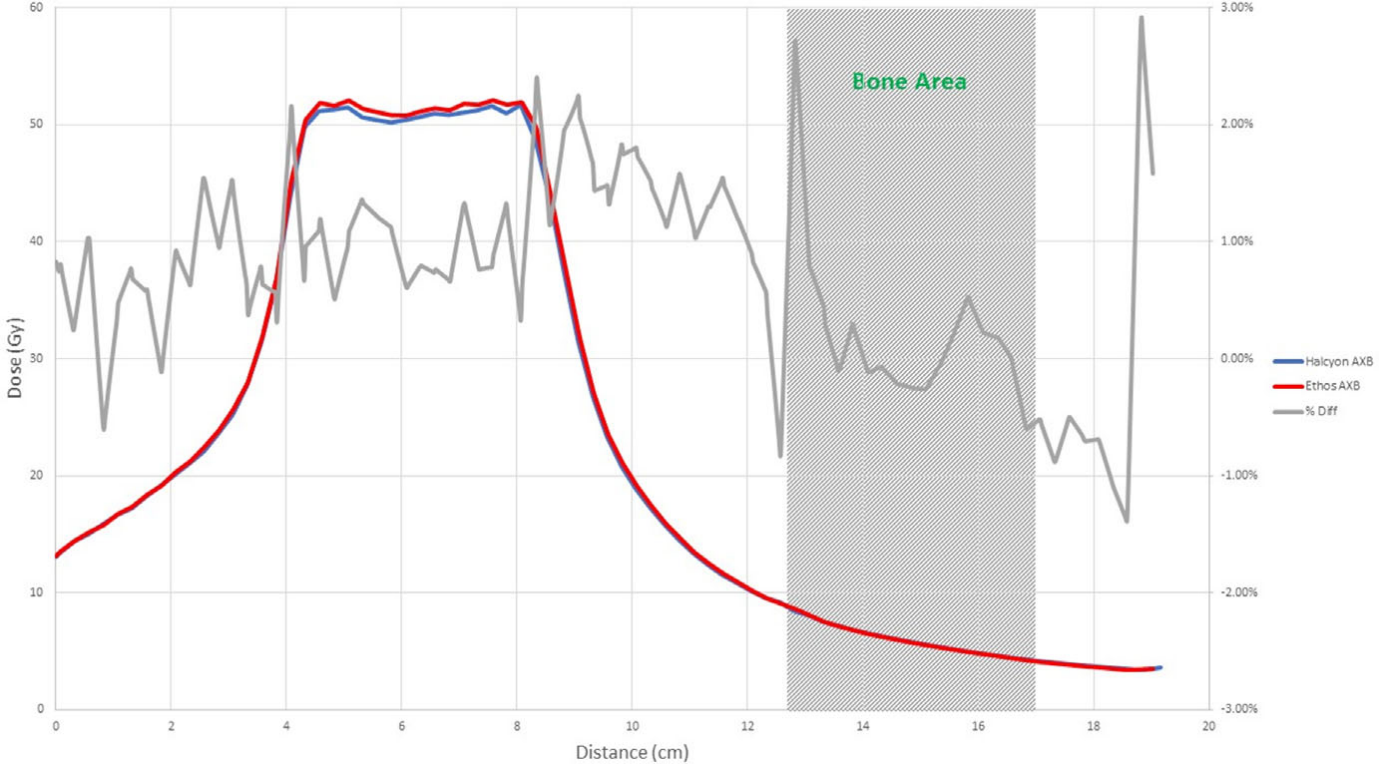
Comparison of the vertical line dose profile of the VMAT plan calculated by the Ethos AXB model and the Halcyon AXB model.

**Fig. 12:**
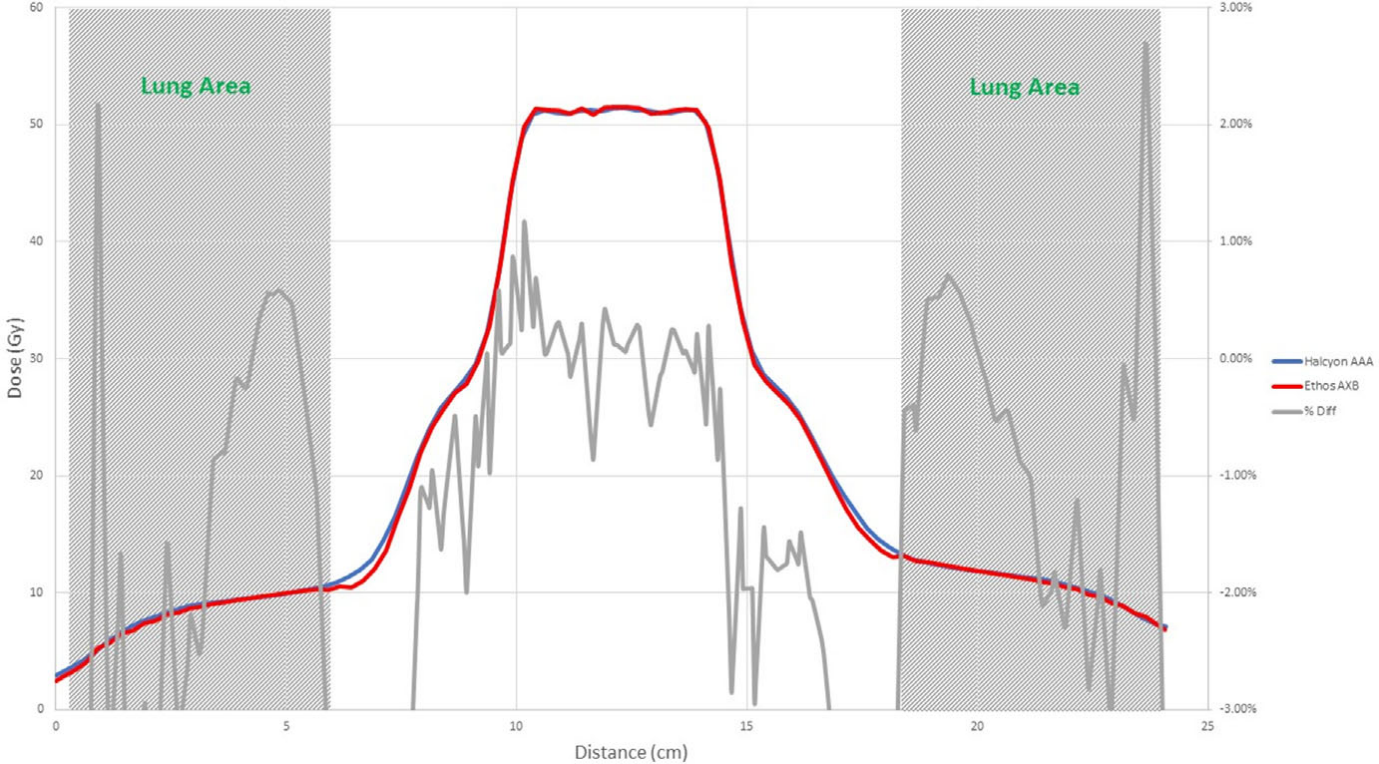
Comparison of the horizontal line dose profile of the IMRT plan calculated by the Ethos AXB model and the Halcyon AAA model.

**Fig. 13:**
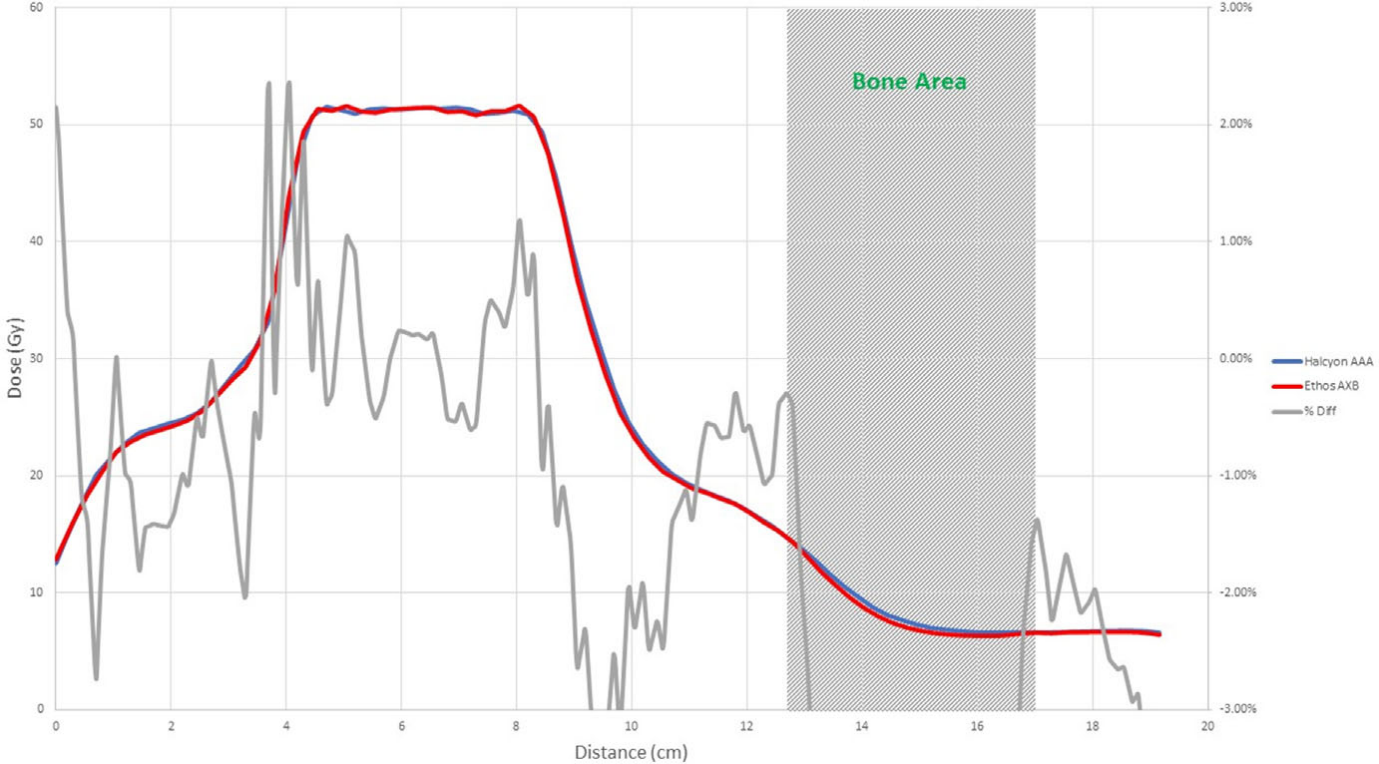
Comparison of the vertical line dose profile of the IMRT plan calculated by the Ethos AXB model and the Halcyon AAA model.

**Fig. 14:**
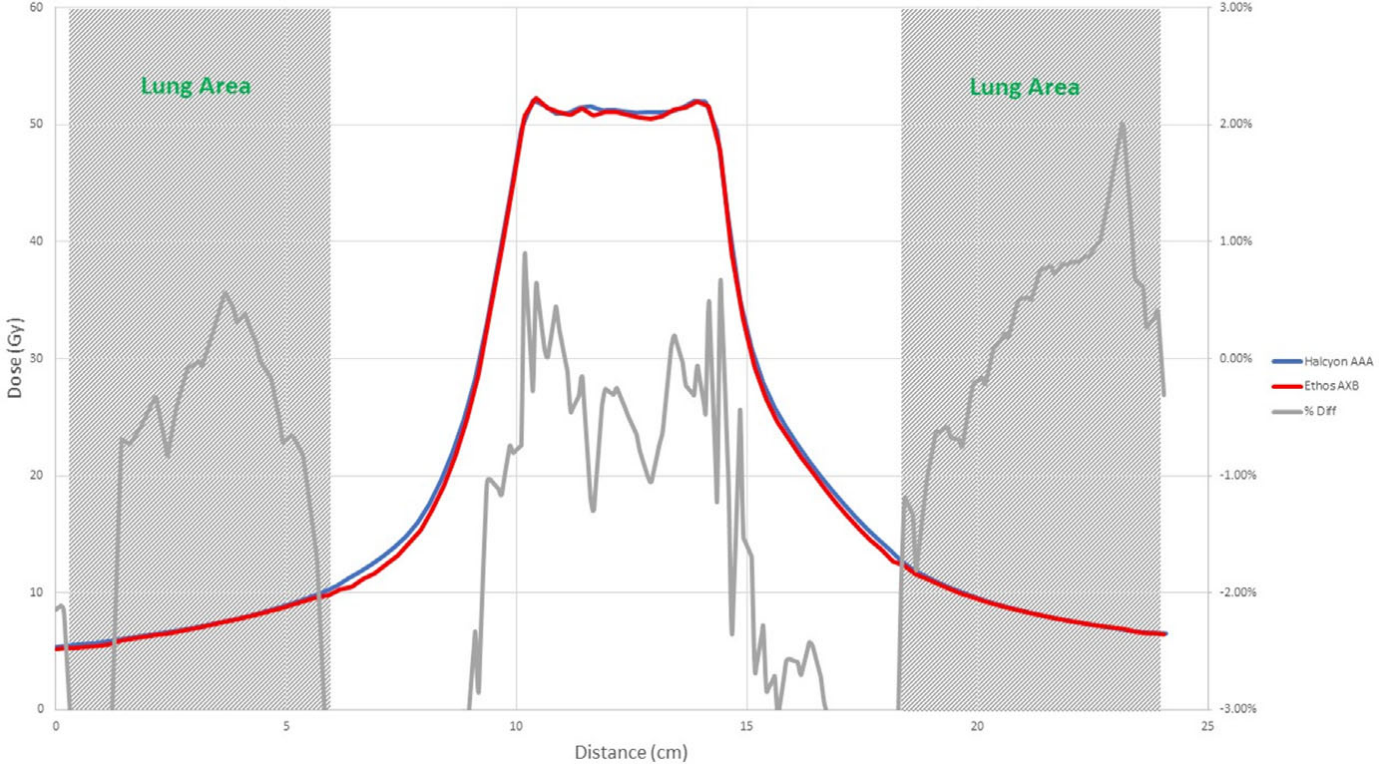
Comparison of the horizontal line dose profile of the VMAT plan calculated by the Ethos AXB model and the Halcyon AAA model.

**Fig. 15:**
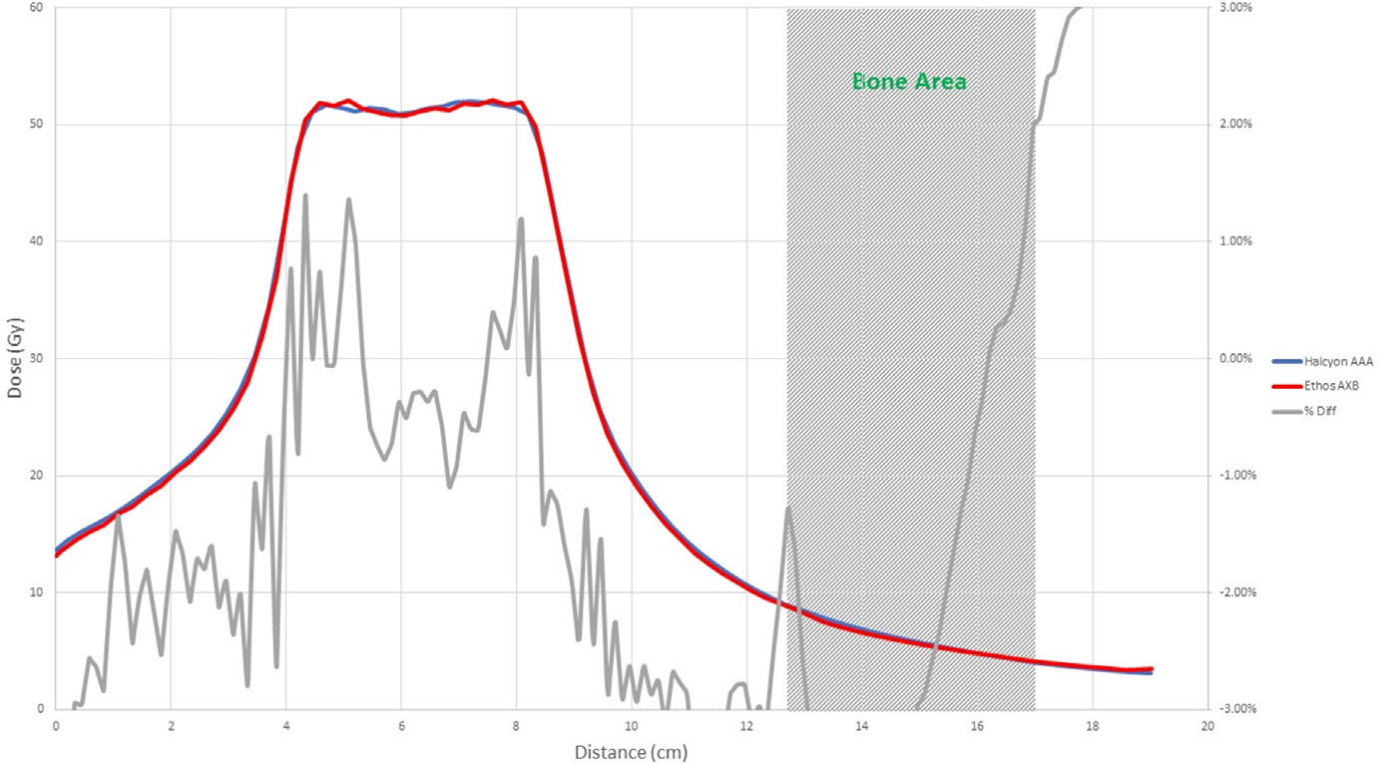
Comparison of the vertical line dose profile of the VMAT plan calculated by the Ethos AXB model and the Halcyon AAA model.

Figures 8, 9, 10, 11, 12, 13, 14, 15 compare line dose profiles calculated by the Ethos AXB model, the Halcyon AXB model, and the Halcyon AAA model. For the two heterogeneity plans, both a horizontal line dose profile that goes through the target and bilateral lungs and a vertical line dose profile that goes through the target and the bone are plotted and compared. Heterogeneity tissue areas (lung/bone) are marked on the figures.

## 4 DISCUSSIONS

### 4.A Basic tests

The accuracy of PDDs and profiles calculated by the preconfigured Ethos AXB beam model was first verified. Results suggested that PDDs calculated by the model were within ±1.5% of measurements beyond the buildup region for field sizes > 1 × 1 cm^2^ and within ±2.0% beyond the buildup region for the field size of 1 × 1 cm^2^. In addition, for all field sizes, the calculated PDDs were slightly higher than the measured PDDs. Similarly, PDDs calculated by the Halcyon AXB model were also slightly higher than measurements, and in this case the discrepancy was larger especially towards the tail of small‐field PDDs. For fields larger than 2 × 2 cm^2^, PDDs calculated by the Halcyon AXB model showed an agreement of ±2.0% beyond the buildup region, whereas the maximum variation between the calculation and the measurement was almost 3.0% at 1 × 1 cm^2^.

As both models utilized the same algorithm, this difference in PDD calculations could only be attributed to variations in the preconfigured models. Although both models utilized identical beam data and evaluation criteria, the modeling processes were different, thereby contributing to differences in the PDDs. During the modeling process, Varian developed and optimized the preconfigured beam models to achieve a maximum of 2 % and 2 mm agreement of 90% of points evaluated between the GBD and the calculated dose.^2^ Although both models may have met these criteria, the Halcyon AXB model clearly shows worse accuracy in PDD matching and may therefore require beam tuning when implemented. However, tuning the energy away from GBD can introduce other complications, potentially hindering beam matching with other Halcyon/Ethos linacs as well as future maintenance.

**Fig. 16:**
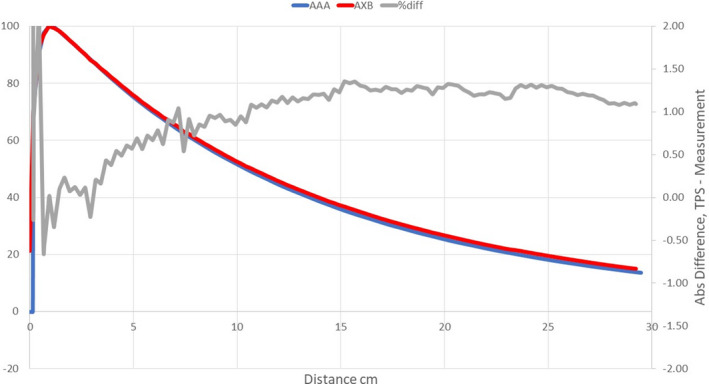
Comparison of the 1 x 1 cm^2^ PDDs calculated by the Ethos AXB model and the Halcyon AAA model. The maximum difference is close to 1.5%. Red: Calculated PDD; Blue: Measured PDD; Grey: % difference.

Alternatively, PDDs calculated by the Halcyon AAA model showed an agreement similar to that of the Ethos AXB model, which was within ±1.5% of measurements beyond the buildup region for field sizes > 1 × 1 cm^2^, and within ±2.0% beyond the buildup region for the field size of 1 x 1 cm^2^. However, for most field sizes, the calculated PDD was slightly lower than the measured PDD. This difference between the Ethos AXB model and the Halcyon AAA model is more obvious when plotted in the same graph (Fig. 16), especially towards the tail of the curve.

Since several studies have suggested that the AAA algorithm and the AXB algorithm demonstrate good agreement in water,^4^, ^5^, ^8^, ^18^ it is likely this difference again arises from variations in the preconfigured models. It can be seen that although both the Ethos AXB model and the Halcyon AAA model have met Varian's modeling criteria, their difference is not negligible.

**Fig. 17:**
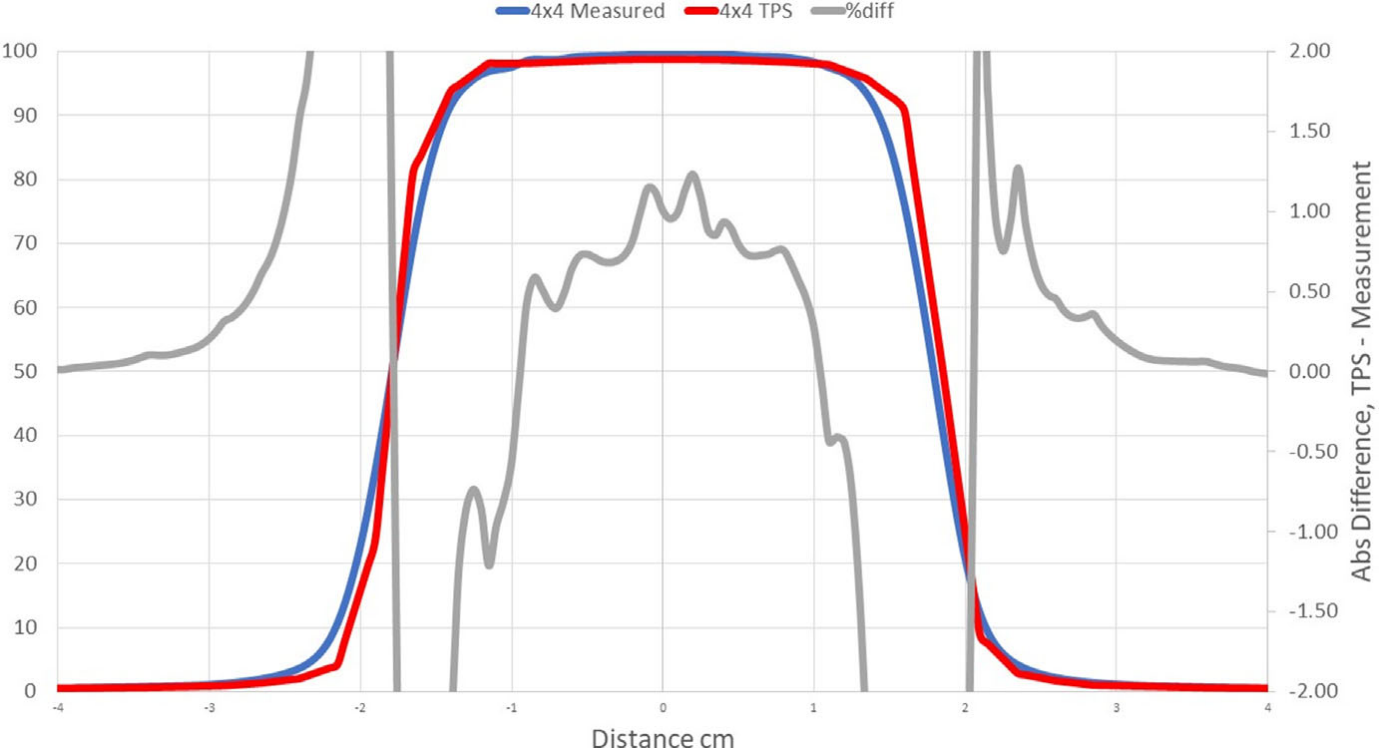
Comparison of the measured profile and the profile calculated by the Ethos beam model at a field size of 4 x 4 cm^2^. In addition to larger differences, shapes of the two profiles were different. Red: Calculated PDD; Blue: Measured PDD; Grey: % difference.

Profile comparisons indicated that for the Halcyon AXB and AAA models, the agreement between the calculated and the measured profiles was between ±1.5% in the umbra region for all field sizes. On the contrary, for the Ethos AXB model, although calculated profiles of larger field sizes (10 × 10 and 28 × 28 cm^2^) showed similar agreement in the umbra region, this was not the case for the 4 × 4 cm^2^ field, where larger differences were observed. In addition, the shape of the calculated profile was slightly wider than that of the measured one, as shown in Fig. 17.

**Fig. 18:**
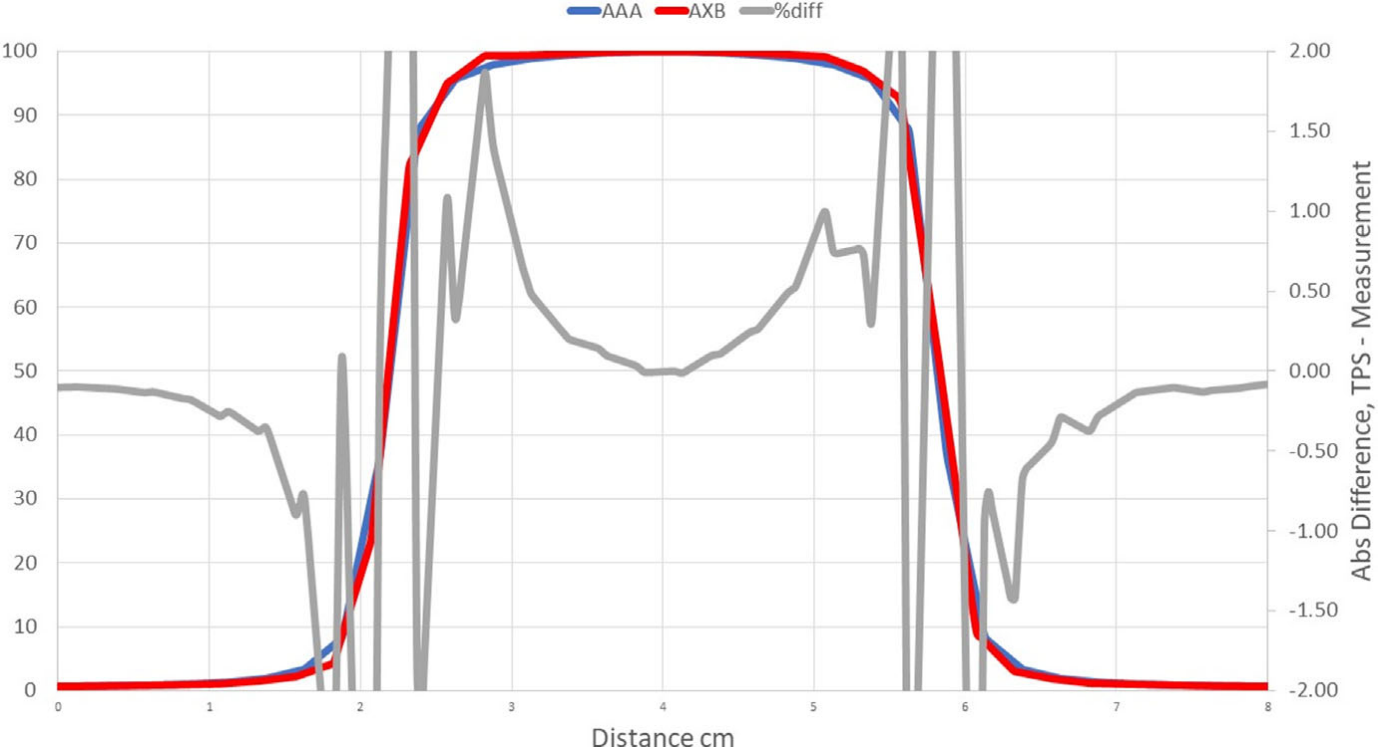
Comparison of the 4 x 4cm^2^ profile calculated by the Ethos AXB model and the Halcyon AAA model, both at a calculation resolution of 2.5 mm. Red: Ethos AXB profile; Blue: Halcyon AAA profile; Grey: % difference.

Further investigation indicated that this difference came from the calculation resolution in the Ethos TPS. The current version of Ethos (Version 1.0) only allows two calculation resolutions: 3.0 and 2.5 mm. Even the smaller resolution (2.5 mm) was too large for small field calculations. Alternatively, all Eclipse PDD and profile calculations were performed with a resolution of 1.0 mm, which led to a substantially improved agreement with measured profiles. To further demonstrate the effect of calculation resolution, the 4 × 4 cm^2^ PDD and profile of the Halcyon AAA model were recalculated in Eclipse with a calculation resolution of 2.5 mm. Although the PDDs did not change significantly, the profiles varied substantially and became similar to that of Ethos, as shown in Fig. 18.

The effect of calculation resolution on the dosimetric accuracy of small fields has been covered by multiple studies^26^, ^27^, ^28^ and is beyond the scope of this paper. However, the fact that the smallest calculation resolution in the Ethos TPS is 2.5 mm significantly limits the accuracy of the system in calculating plans involving small targets. It is assumed that smaller calculation resolutions are disabled to accelerate the calculation speed of online adaptive plans, but when implementing the system, users should be aware of the effect of the calculation resolution on small targets. The restricted calculation resolution also represents a significant challenge in implementing stereotactic treatments on the Ethos platform.

Results of output factors in water showed that all three models had good agreement with measurement. For the Ethos AXB model, the output factors of both square and elongated fields were within ±1.5% of measurements, with the largest difference being −0.9% at 2 × 2 cm^2^ and 3 × 28 cm^2^. For the Halcyon AXB model, the largest difference was −1.2% at 3 × 28 cm^2^, while the output factors of all other field sizes were within ±1.0% of measurements. For the Halcyon AAA model, the output factors of fields were within ±2.0% of measurements, with the largest difference being −1.7% at 2 × 2 cm^2^. Although output factors calculated by the Halcyon AAA model showed larger differences than those calculated by the Ethos AXB model and the Halcyon AXB model, all three were acceptable for clinical use.^29^ In addition, pairwise comparisons indicated that differences between these three models were not statistically significant (*P* > 0.05). Interestingly, for all three models, larger deviations were observed at the 2 × 2 cm^2^ and the 3 × 28 cm^2^ fields, which could be an intrinsic modelling issue of preconfigured Halcyon/Ethos models.

### 4.B Advanced tests for specific planning or treatment conditions

To test the performances of both models in calculating IMRT/VMAT in plans in a homogeneous material, the chair test and a set of clinical plans were utilized, with measurements acquired using both a single‐point method and a 3D method.^22^ In the chair test, the Halcyon AXB model showed the best agreement with measurements, where all differences were within ±0.5% regardless of how complex the plan was. This was followed by the Ethos AXB model, the differences of which were within ±1.0% of measurements. The Halcyon AAA model demonstrated the largest variations, up to 2.3% at the most complex plan. A test of significance could not be performed as the number of samples was small.

Chair test is a commonly used test that checks the dosimetric accuracy of the entire dynamic multi‐leaf‐collimator (dMLC) system by introducing thin MLC apertures and high plan complexity.^30^ Since the chair test in this paper was conducted on a homogeneous water phantom and the calculation resolution was kept the same (2.5 mm), differences introduced by different algorithms and calculation resolutions were negligible. Therefore, the fact that both the Ethos AXB model and the Halcyon AXB model presented a considerably better point dose agreement than the Halcyon AAA model was most likely because of a better modelling accuracy in small fields. This was consistent with findings of the previous section, where both the Ethos AXB model and the Halcyon AXB model demonstrated better agreement in output factors of small (2 × 2 cm^2^) and thin (3 × 28 cm^2^) fields. Interestingly, only the Ethos AXB model displayed a linear correlation between plan complexity and measurement difference (R^2^ = 0.915) but not the other two models (R^2^ = 0.347 for the Halcyon AXB model and 0.004 for the Halcyon AAA model). However, the sample size was too small to be conclusive of this correlation.

It can be seen from Tables 4 and 5 that all three models displayed good consistency with measurements in homogeneous phantoms in both point doses and 3D doses, indicating they were capable of computing complex IMRT and VMAT plans in the clinical environment. The Ethos AXB model and the Halcyon AXB model showed similar agreement, whereas the Halcyon AAA model demonstrated slightly worse agreement. When dose calculations from these three models were directly compared, the agreement was consistently 100.0% under a gamma criterion of 3% and 2 mm, as shown in Table 6.

These results indicated that the performance of the Ethos AXB model in calculating common clinical plans were comparable to those of the Halcyon models. In addition, a consistent trend where the AXB models demonstrated a slightly improved agreement than the AAA model was observed, which was most likely due to a better accuracy in modeling small and thin fields. Regardless, the accuracy of all three models was considered clinically acceptable.^22^ This finding is consistent with previous literature, that if optimal settings were applied in the algorithm configuration phase, both the AXB and the AAA algorithms showed satisfactory accuracy in calculating IMRT and VMAT plans.^8^, ^31^, ^32^

### 4.C Advanced tests to investigate the management of tissue heterogeneity

To determine the absorbed dose in the irradiated tissues more accurately, dose calculation algorithms must account for heterogeneity.^33^ Differences of the AXB and AAA algorithms in calculating doses in heterogeneous tissues have been covered in multiple studies. For example, Bush et al.^3^ compared the dosimetric accuracy of AXB with both Monte Carlo (MC) and AAA, and found that for 6 and 18 MV photon beams, the agreement between MC and AXB was ±3.0%, while that between MC and AAA could be up to 17.5%. In addition, this difference between AAA and AXB was most obvious in air/tissue or tissue/metal interfaces. For example, Kan et al.^31^ demonstrated that AAA overestimated doses near air/tissue interfaces in an anthropomorphic phantom by up to 10%, while for AXB this was within 3%. Similarly, Rana et al.^34^ comprehensively analyzed the dosimetric accuracy of AXB and AAA beyond different sizes of air gap in simple geometric circumstances, and suggested that the discrepancies between AXB and measured data seen were from −3.8% to 0.9%, whereas the AAA differences with measurement were from −3.1% to −10.9%. Based on the results, they concluded that AXB was more appropriate to use for dose calculations when low‐density heterogeneities were involved. Other studies^35^, ^36^, ^37^ have drawn similar conclusions, that although both the AXB and the AAA algorithm can meet the RTOG 0813 dosimetric criteria, in general, AXB presented an improved dosimetric accuracy in the presence of inhomogeneity.

The Ethos TPS uses the same AXB algorithm in Eclipse (albeit with a different architectural implementation as well as a different preconfigured model), but only reports dose to medium.^2^ Since the purpose of this study was not to provide another validation of the AXB algorithm, but rather the preconfigured Ethos beam model calculated with the AXB algorithm in the Ethos TPS, only a simple test was conducted to investigate the model’s management of tissue heterogeneity. In chamber measurements, all three models showed an agreement within ±2.0% of the measurement, suggesting that they were acceptable for clinical use. The number of samples was too small for a test of significance, but in general all three models presented similar point dose agreement, with that of the AAA model slightly worse than the AXB models.

In addition to the chamber measurements, which was located in a homogeneous insert that was relatively distant from any heterogeneous tissues, line dose profiles going through heterogeneous tissues were compared between the three models. Profile comparisons between the Ethos AXB model and the Halcyon AXB model indicated that the two models generally agreed well in both homogeneous and heterogeneous tissues as well as tissue interfaces, with local differences less than ±3.0% in high‐dose areas. However, despite using the same algorithm, the two models were still showing minor variations in both directions regardless of the plan type, although these variations were clinically insignificant. This finding suggested that although the Ethos TPS adopted the same AXB algorithm that had been previously validated in Eclipse, due to a different computation environment and modelling processes, an independent validation was still required to verify the accuracy of the algorithm was adequate for the clinical scope.

Profile comparisons between the Ethos AXB model and the Halcyon AAA model indicated that for both the IMRT and the VMAT plans, while agreement in water‐equivalent tissues was mostly within ±2.0%, in the tissue/lung interface and bone area substantially larger differences of over ±5.0% were seen. Since the difference was not consistent across all areas, but rather confined to either heterogeneous tissues or tissue interfaces, it was most likely due to differences in the algorithm. It can also be seen that the Halcyon AAA model tends to predict higher doses in the lung/tissue interface, which is consistent with previous literature.^31^, ^34^

Although the tissue heterogeneity test in this paper was relatively simple, results consistent with previous studies were seen, which suggested that the Ethos AXB model demonstrated an improved dosimetric accuracy in the presence of inhomogeneity than the Halcyon AAA model. Although the Ethos AXB model and the Halcyon AXB model adopted the same algorithm, minor variations were observed across dose profiles, indicating that the two models were not identical. Therefore, an independent verification should always be performed when an existing algorithm is implemented in a new computing environment.

## 5 CONCLUSION

In this study, to validate the clinical use of dose calculations performed with the preconfigured Ethos AXB model, calculations in various conditions were conducted in the Ethos TPS. The calculated results were subsequently compared to measurements as well as dose calculations from the Halcyon AXB and AAA models, which have been verified by both literature and previous commissioning tests. Results indicated that the Ethos AXB model demonstrated a comparable if not superior dosimetric accuracy to the Halcyon AXB model in basic and complex calculations. The Ethos AXB model also demonstrated superior dosimetric accuracy in modulated and heterogeneous plans when compared to the Halcyon AAA model due to differences in the algorithm. Despite the fact that the same algorithm was utilized, the Ethos AXB model and the Halcyon AXB model still exhibited variations across a range of tests, although these variations were predominantly insignificant in the clinical environment.

Ethos is a novel on‐line radiotherapy treatment platform that was recently released by Varian. By incorporating artificial intelligence, it is capable of creating plans automatically at a high speed and comes with a preconfigured model. However, the introduction of a brand‐new TPS onto a new architectural platform and the lack of human interaction in beam modeling and plan creation means that clinical physicists must comprehensively verify the accuracy of the system before releasing it for clinical use. In this study, we comprehensively validated the preconfigured Varian Ethos AXB beam model for treatment planning dose calculations. On the basis of this study, clinical physicists can perform data validation instead of a full data commissioning when implementing the Ethos system, thereby adopting a more efficient approach for Ethos installation.

## CONFLICT OF INTEREST

The authors declare that there is no duality of interest that they should disclose.
